# Effect of Soy Protein Isolate on the Quality Characteristics of Silver Carp Surimi Gel during Cold Storage

**DOI:** 10.3390/foods13152370

**Published:** 2024-07-26

**Authors:** Songxing Zhang, Zeyu Song, Junhao Gu, Xueqian Guo, Yangling Wan, Huaixiang Tian, Xichang Wang

**Affiliations:** 1 College of Food Science and Technology, Shanghai Ocean University, Shanghai 201306, China; zhsx999@163.com (S.Z.); szy1963959735@163.com (Z.S.); gujunhao202109@163.com (J.G.); 2 Shanghai Engineering Research Center of Aquaculture, Shanghai Ocean University, Shanghai 201306, China; 3 Shanghai Engineering Research Center of Aquatic-Product Processing and Preservation, Shanghai 201306, China; 4 Department of Food Science & Technology, School of Agriculture & Biology, Shanghai Jiao Tong University, Shanghai 201306, China; guoxueqian@sjtu.edu.cn; 5 Wilmar Shanghai Biotechnology Research and Development Center Co., Ltd., Shanghai 200120, China; wanyangling@cn.wilmar-intl.com; 6 School of Perfume and Aroma Technology, Shanghai Institute of Technology, Shanghai 201418, China

**Keywords:** surimi products, soy protein isolate, composite gel, gel quality

## Abstract

This study mainly investigated the effect of soy protein isolate (SPI) on the gel quality of silver carp surimi under different storage conditions (storage temperatures of 4 °C, −20 °C, and −40 °C, and storage times of 0, 15, and 30 d). The results found that 10% SPI could inhibit the growth of ice crystals, improve the water distribution, enhance the water holding capacity of the gels, and strengthen the interaction between surimi and proteins. Compared to the control group, the composite silver carp surimi gel exhibited superior quality in texture, chemical interactions, and rheological properties during cold storage. Fourier transform infrared spectroscopy revealed an increasing trend in α-helix and β-turn content and a decreasing trend of β-sheet and random coil content. As storage time increased, the gel deterioration during cold storage inhibitory effect of the treatment group was superior to the control group, with the best results observed at −40 °C storage conditions. Overall, SPI was a good choice for maintaining the quality of silver carp surimi gel during cold storage, which could significantly reduce the changes in the textural properties during cold storage with improved water holding capacity.

## 1. Introduction

Surimi products are becoming more and more popular among consumers due to their nutritional value, diverse flavors, and low cholesterol content. Most surimi products available in the market are designed for room temperature consumption. However, to better meet consumer demands for freshness and extend the shelf life, cold storage for surimi products is often necessary. During the cold storage process, changes in the water state of the surimi occur due to differences in freezing temperatures and rates. These changes may lead to the denaturation of myofibrillar proteins, ultimately resulting in a decline in the quality of surimi products [1,2,3,4].

To address the issue of quality degradation during cold storage and improve the quality of chilled surimi products, various plant-based materials such as starches, dietary fibers, and plant-based proteins have been added to surimi [5,6,7]. Researchers found that the quality of starch-added surimi gels was related to the particle size of the starch, and the water absorption capacity and structure deteriorated significantly after freezing. Starch with small particle sizes also significantly influenced the quality changes of heated surimi gels after freezing and thawing. Cao et al. [8] found that inulin could alter the water distribution in surimi, reduce the freezing point, inhibit the growth of ice crystals, and enhance the interaction between surimi and proteins. Hasanpour et al. [9] found that soy protein concentrate and xanthan gum could improve the water holding capacity and gel-forming ability of surimi during frozen storage. Zhang et al. [10] evaluated the influence of various freezing methods on the gel properties of grass carp surimi and found that the pressure shift freezing treatment could significantly improve the quality of the surimi gels. Yang et al. [11] studied the effects of different freezing methods, including raw-freezing-setting-heating, raw-setting-freezing-heating, and raw-setting-heating-freezing, on the quality changes of surimi gels, and revealed that the raw-setting-heating-freezing method provided better resistance against the impact of ice crystals of fish tissue during the frozen period, while the gel formed during the setting process delayed the erosion of ice crystals of the fish tissue.

SPI is a plant-based protein that enhances health-enhancing properties and cardiovascular-protective effects [12,13], representing a cost-effective and nutritionally comprehensive natural resource with a comprehensive amino acid composition. SPI has excellent processing and functional characteristics [14] and is widely used in food processing due to its high content of sulfur-containing amino acids in the globulin molecular chains. Such characteristics are beneficial to the formation of a fibrous network structure. Under the action of heat and shear, the SPI chains can easily open up, exposing molecular binding sites, which can further oxidize to form disulfide bonds [15]. Liu et al. [16] found that the addition of SPI as a binder in meat products could not only improve the quality and yield but also reduce production costs. You et al. [17] found that the textured SPI can increase the supply of fish protein, which was conducive to the improvement of the gel strength of fish intestines with acceptable quality after 120 d of storage at −18 °C. Xu et al. [18] found that the co-stabilized emulsions of SPI and κ-carrageenan could enable the uniform distribution of small oil droplets within the dense and ordered protein network of surimi gels, thereby optimizing the gel properties of the surimi gels. However, the specific impact of SPI on the water retention and textural changes of surimi gels during cold storage remains unclear.

In this study, SPI was added into the silver carp surimi to investigate the impact of SPI on the water retention and textural changes of the composite gels during storage under different conditions (storage temperatures of 4 °C, −20 °C and −40 °C, and storage time of 0, 15, and 30 d). A comprehensive evaluation of the quality changes in the composite gels during cold storage was conducted through various techniques, including ice crystal morphology determination, water distribution analysis, water holding capacity, texture analysis, gel strength, Fourier transform infrared spectroscopy, total sulfhydryl content, chemical interactions, rheological properties, scanning electron microscopy, color analysis, and sodium dodecyl sulfate polyacrylamide gel electrophoresis. This research aims to provide theoretical support for the cold storage of surimi products, enabling a deeper understanding of the changes that occur in composite gels containing SPI during cold storage.

## 2. Materials and Methods

### 2.1. Materials

Frozen minced silver carp (76.65% moisture) was purchased from Jingzhou Honghu Jingli Aquatic Foods Co., Ltd. (Honghu, China) and transported to Shanghai Ocean University via cold chain logistics. SPI was obtained from Yihai Kerry Arawana Holdings Co., Ltd. (Shanghai, China). Chemical reagents of analytical grade including sodium chloride, urea, Tris, hydrochloric acid, glutaraldehyde solution, formalin solution, ethanol, eosin, EDTA, sodium dodecyl sulfate (SDS), and silicone oil were all purchased from Sinopharm Chemical Reagent Co., Ltd. (Shanghai, China).

### 2.2. Preparation of Surimi Gels

Frozen silver carp surimi was thawed at 4 °C for 6 h. After cutting into small pieces, silver carp surimi fragments were put into a meat grinder (QSJ-B03E1, bear, Fusan, China) and shredded for 1 min. Then, 2.5% sodium chloride was added to the surimi and stirred for 1 min. An aliquot of homogenized SPI was then added into the above-mentioned stirred surimi (10%) [19], the final moisture content was adjusted to 80%, and stirring was performed for 1 min. The abovementioned sample was loaded into a polyethylene casing with a diameter of 40 mm and then heated in a water bath at 40 °C for 30 min and 90 °C for 30 min. Finally, the samples were cooled in an ice-water bath for 30 min. The samples were stored in refrigerators at 4 °C (Haier, Qingdao, China, Model SC-412), −20 °C (Haier, Model DW-40L348J) and −40 °C (Haier, Model DW-30L508), respectively. All the frozen samples were placed in a refrigerator at 4 °C to thaw before further analysis.

Control-PS represents the fresh gel sample of pure surimi gel. PS-4-15 indicated that the pure surimi gel was frozen at 4 °C for 15 d. PS-4-30 indicated that the pure surimi gel was frozen at 4 °C for 30 d. PS-20-15 indicated that the pure surimi gel was frozen at −20 °C for 15 d. PS-20-30 indicated that the pure surimi gel was frozen at −20 °C for 30 d. PS-40-15 indicated that the pure surimi gel was frozen at −40 °C for 15 d. PS-40-30 indicated that the pure surimi gel was frozen at −40 °C for 30 d. Control-SPI represents the fresh gel sample of SPI-surimi gel. SPI-4-15 indicated that SPI-surimi composite gel was frozen at 4 °C for 15 d. SPI-4-30 indicated that SPI-surimi composite gel was frozen at 4 °C for 30 d. SPI-20-15 indicated that SPI-surimi composite gel was frozen at −20 °C for 15 d. SPI-20-30 indicated that SPI-surimi composite gel was frozen at −20 °C for 30 d. SPI-40-15 indicated that SPI-surimi composite gel was frozen at −40 °C for 15 d. SPI-40-30 indicated that SPI-surimi composite gel was frozen at −40 °C for 30 d.

### 2.3. Ice Crystal Observation

Ice crystal observation was carried out according to the method described by Zhang et al. [20] with some modifications. To visualize the ice crystals within the surimi, eosin staining was employed. Initially, the surimi samples were sliced into 5 μm-thick sections under frozen conditions at −20 °C. Subsequently, the surimi sections were fixed in a 37% (*v*/*v*) formalin solution for 2 h, and then dehydrated with 50% (*v*/*v*) ethanol for 0.5 h before stained with eosin. The stained samples were further dehydrated using 70%, 80%, 90%, 95%, and 100% (*v*/*v*) ethanol in turn. Finally, the ice crystal morphology of the surimi sections was observed under a 40-fold optical microscope (BA410, Motic Group Co., Ltd., Chengdu, China).

### 2.4. Low-Field NMR (LF-NMR)

The water distribution analysis was carried out according to the method described by Wei et al. [21] with minor modifications. The surimi samples were cut into cylinders with a height of about 2 cm using a sampler and then placed in the nuclear magnetic resonance (NMR) instrument (Shanghai Electronic Technology Co., Ltd., Shanghai, China, PQ-001). The Carr-Purcell-Meiboom-Gill (CPMG) pulse sequence was used to measure the spin-spin relaxation time T_2_. The acquired data were inverted using the software provided with the NMR instrument, resulting in the extraction of T_2_ values and the corresponding peak areas and area ratios.

### 2.5. Water Holding Capacity (WHC)

The WHC was carried out according to the method described by Yang et al. [22] with some modifications. The gel samples were meticulously sectioned into consistently thin slices (approximately 5 mm in thickness). The slices were then precisely weighed (W1) and were enveloped in a triple layer of filter paper. Subsequently, the wrapped samples were transferred into a 50 mL tube and centrifuged at 4 °C and 5000 rpm for 5 min (H1850R Cence, Changsha, China) and weighed again (W2). The weight loss was calculated using the following formula:

(1) $$\mathrm{WHC}\;(\%)=1-\frac{W1-W2}{W1}\times 100\%$$

### 2.6. Texture Profile Analysis (TPA)

TPA was carried out according to the method described by Luo et al. [23] with minor modifications. A TA-XT plus texture analyzer (Stable Micro Systems, Surrey, UK) equipped with a P/50 probe was used, the probe speed was set at 1.0 mm/s, and 8 measurements were taken for each sample. The obtained data were expressed as hardness (g), springiness (mm), chewiness (g), and resilience.

### 2.7. Gel Strength

Gel strength was measured based on the method proposed by Zhang et al. [24]. A TA-XT plus texture analyzer equipped with a P/5S probe (Stable Micro Systems, Surrey, UK) was used. The gel samples (2.0 cm in height) were restored to room temperature before testing. The probe speed was set at 1.0 mm/s. Each measurement was repeated 8 times. The gel strength was calculated as follows:

Gel strength (g·mm) = Breaking force (g) × Deformation (mm)(2)

### 2.8. Fourier Transform Infrared Spectroscopy (FTIR) 

FTIR was measured based on the method proposed by Zhao et al. [25]. Briefly, the freeze-dried gel samples (FD-1C-50, Boyikang, Beijing, China) were detected on the Spotlight 400 ATR-FTIR spectrometer (PerkinElmer Company, Waltham, MA, USA). The FTIR spectra were recorded at the wavenumber range of 4000–400 cm^−1^. The amide I band (1700–1600 cm^−1^) was analyzed by the PeakFit software version 4.12 (Systat Software Inc., San Jose, CA, USA).

### 2.9. Determination of Sulfhydryl Group Content

The total sulfhydryl group was measured based on the method proposed by Jiang et al. [26] with minor modifications. First, 0.5 mL of 5 mg/mL protein solution was mixed with 4.5 mL buffer A (10 mmol/L EDTA, 8 moL/L urea, 1% SDS, 0.2 mol/L Tris-HCl, pH 8.0). Then, 4 mL of the mixture was taken out and mixed with 0.5 mL buffer B (1 g/L DTNB, 0.2 mol/L Tris-HCl, pH 8.0). After thoroughly mixing, the mixture was incubated at 40 °C for 30 min and then cooled to room temperature. The absorbance was measured at 412 nm. The control group used 0.6 mol/L NaCl instead of myofibrillar protein. The molar extinction coefficient was 13,700 (mol/L)^−1^ cm^−1^. The total sulfhydryl concentration was calculated according to the following formula:

(3) $$\mathrm{SH}\;(\mathrm{mol}/10^{5}g)=\frac{A\times D}{C\times B}$$

where A represents the absorbance of the mixture, B represents protein concentration, C represents the molar extinction coefficient (13,700 (mol/L)^−1^ cm^−1^) and D represents the dilution ratio.

### 2.10. Chemical Forces

The chemical forces in the prepared gel samples were analyzed by referring to the method of Zhao et al. [27] with slight modifications. Briefly, 2.0 g of gel sample was mixed with 10 mL of different solutions: 0.05 mol/L NaCl solution (SA), 0.6 mol/L NaCl solution (SB), 0.6 mol/L NaCl + 1.5 mol/L urea (SC), 0.6 mol/L NaCl + 8 mol/L urea (SD). The obtained solution was homogenized for 2 min, stored at 4 °C for 6 h, and centrifuged at 12,000 rpm for 10 min. Non-specific linkages between protein molecules in the gel were determined by the protein content in the SA solution. Ionic bonds were represented by the difference in protein content between SB and SA solutions. The contribution of hydrogen and hydrophobic bonds in the gels was estimated using the difference in protein content between SC and SB solutions as well as between SC and SD solutions.

### 2.11. Dynamic Rheological Properties

The dynamic rheological properties of the gel samples were determined by the method proposed by Zhao et al. [27] with slight modifications. AR 2000 ex rheometer (TA Instruments, Newcastle, DE, USA) equipped with a 40 mm parallel steel plate was used to study the dynamic rheological properties of surimi paste. The gap was set at 1 mm. The gel sample was loaded on the stage, and silicone oil was used to prevent water evaporation. During the temperature scanning process, the samples were heated from 20 °C to 90 °C at a ramp rate of 2 °C/min under an oscillatory stress of 2 Pa and a frequency of 1 Hz. The storage modulus (G′, representing elastic properties) and loss modulus (G″, representing viscous properties) were recorded.

### 2.12. Color

The gel color was tested according to the method proposed by Zhao et al. [27] with minor modifications. A colorimeter (Shanghai INESA Physico-Optical instrument Co., Ltd. WSC-S, Shanghai, China) was used to determine gel color. The whiteness calculation formula was as follows [28]:

Whiteness = 100 − [(100 − L*)^2^ + a*^2^ + b*^2^]^1/2^ (4)

where L* represents luminance; a* represents red-green color, b* represents yellow-blue color and whiteness value.

### 2.13. Sodium Dodecyl Sulfate-Polyacrylamide Gel Electrophoresis (SDS-PAGE)

Protein changes were detected by using SDS-PAGE [29]. A 10% acrylamide separation gel and a 5% acrylamide stacking gel were first prepared. Samples (3 mg/mL protein) were dissolved in buffer (5% SDS, 25% glycerol, 10% β-mercaptoethanol, 0.06 M Tris) in a 4:1 ratio and heated in a melting bath at 100 °C for 5 min. Aliquots of 10 μL of liquid chromatography were added to each gel channel. After electrophoresis, the gels were stained with 0.1% (*w/v*) Kaomas blue R250 (distilled water, methanol, and glacial acetic acid 9:9:2) for 30 min and destained in a mixture of distilled water, methanol, and glacial acetic acid (8:1:1) for 2 h.

### 2.14. Statistical Analysis

All measurements were repeated at least three times, and the results were expressed as mean ± standard deviation. Analysis of variance was performed using SPSS 20.0 (SPSS Inc., Chicago, IL, USA), and *p* < 0.05 was considered significant. The chart was drawn using Origin 2023 software (OriginLab Corporation, Northampton, MA, USA).

## 3. Result and Discussion

### 3.1. Growth of Ice Crystals

**Figure 1:**
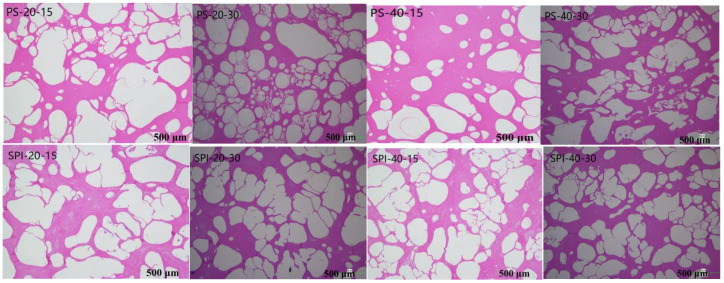
Changes in gel ice crystal morphology under different cold storage conditions (Magnification: 40×).

The growth of ice crystals can easily cause mechanical damage to the sarcoplasmic reticulum of fish, enlarging the myofibrillar spaces and leading to degradation in the texture, color, and nutritional value of products [30]. Figure 1 illustrates the changes in ice crystal morphology in gels under different storage conditions. In the control group, the ice crystals were large and irregular, occupying a significant amount of space and compressing the fiber structure, causing severe damage to the muscle fibers. Under the same conditions, ice crystal particles at −20 °C were larger and more irregular compared to those at −40 °C. In contrast, the treatment group exhibited smaller and more regularly shaped ice crystals, which may preserve the integrity of the muscle tissue.

The abovementioned observation may be attributed to the interaction between SPI and surimi protein gels. The gel-forming properties of SPI likely increased the amount of immobilized water, which may inhibit the nucleation and growth of ice crystals. The extent of mechanical damage to surimi depended on the shape and size of the ice crystals, indicating that controlling the size, shape, and distribution of ice crystals in surimi may have a positive effect on mitigating freeze-induced damage [31].

### 3.2. LF-NMR

**Figure 2:**
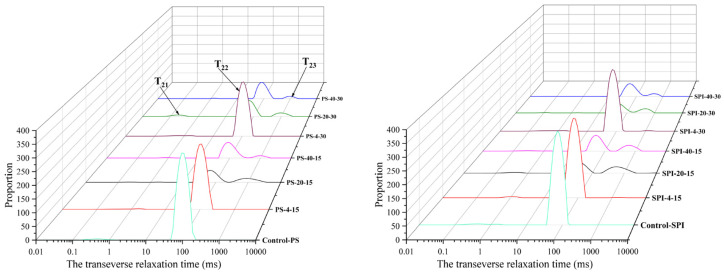
Moisture distribution of gel under different cold storage conditions.

LF-NMR technology undoubtedly holds promising application prospects in predicting the quality characteristics and shelf life of aquatic products [32]. As shown in Figure 2, the varying trends of water distribution in gels under different cold storage durations and temperatures are depicted through the LF-NMR curves of the gel samples, revealing four distinct peaks. Among these peaks, T_21_ represents the tightly bound water associated with proteins, T_22_ corresponds to the immobilized water within the protein network structure, and T_23_ indicates the free water within the gel network [33].

In the comparison between the control group and the treatment group in samples, it was observed that the T_22_ value in the treatment group increased significantly. Furthermore, statistical analysis demonstrated a significant rise in the T_22_ peak for the treatment group compared to the control group (*p* < 0.05). This observation can be attributed to the gel-forming properties of SPI, which enhance the WHC of the composite gel [19].

For the 4 °C storage, both the control group and the treatment group showed a decreasing trend in the T_22_ value of their gels at 15 and 30 d of cold storage. Compared to the control group, the treatment group exhibited a slower decline in T_22_ at both 15 and 30 d of cold storage, indicating that the addition of SPI significantly altered the T_22_ properties of the composite gel.

For the −20 °C storage, the T_22_ value of the gel in the control group decreased significantly from 0 to 30 d (*p* < 0.05). Meanwhile, the T_23_ value of the control group showed an increasing trend at both 15 and 30 d of cold storage (*p* < 0.05). For the treatment group’s gel, the T_22_ value also decreased significantly after 15 and 30 d of storage, while the T_23_ value demonstrated an increasing trend at the same time points.

For the −40 °C storage, the gel T_22_ of the control group and the treatment decreased significantly after 15 and 30 d of cold storage (*p* < 0.05). T_23_ showed an increasing trend at 15 and 30 d. However, T_23_ in both groups showed an increasing trend after cold storage for 15 and 30 d. Compared with the control group, the T_22_ of the treatment group showed a slow increase after 15 d of cold storage, but showed a downward trend after 30 d of cold storage. In addition, T_23_ showed an increasing trend after cold storage for 15 and 30 d.

The results indicate that the treatment groups exhibited higher T_22_ content at all three cold storage temperatures. The addition of SPI slowed down the decline in T_22_ and reduced the increase in T_23_ during the cold storage process. This may be attributed to the gel-forming properties of SPI, which enhance the water binding capacity. Under the same conditions, the lower the freezing temperature, the shorter the cold storage time, and the slower the LF-NMR change. The affinity of myofibrillar proteins for water primarily depends on the exposed hydroxyl and carboxyl groups as well as other amino acid functional groups in their spatial structure [34]. Water with longer relaxation times exhibits looser binding to macromolecules and greater mobility [35]. As the storage time progressed, the relaxation times of all groups increased, indicating an increase in water mobility. Temperature fluctuations induced structural changes in myofibrillar proteins, reducing their ability to bind water [36].

In fresh samples, there was no statistically significant difference in the T_21_ relaxation times among the various groups (*p* > 0.05). This may be due to the tight binding of bound water to protein macromolecules, and general cold storage treatments have minimal impact on bound water. However, the T_21_ relaxation times gradually increased in all groups as the cold storage duration extended. The increase in T_21_ during cold storage may be attributed to the exposure of hydrophobic groups, leading to the unfolding of protein structures. This reduces the protein’s ability to bind water, thereby increasing the mobility of bound water [37]. These findings are consistent with the observed trends in ice crystal morphology.

### 3.3. WHC

**Figure 3:**
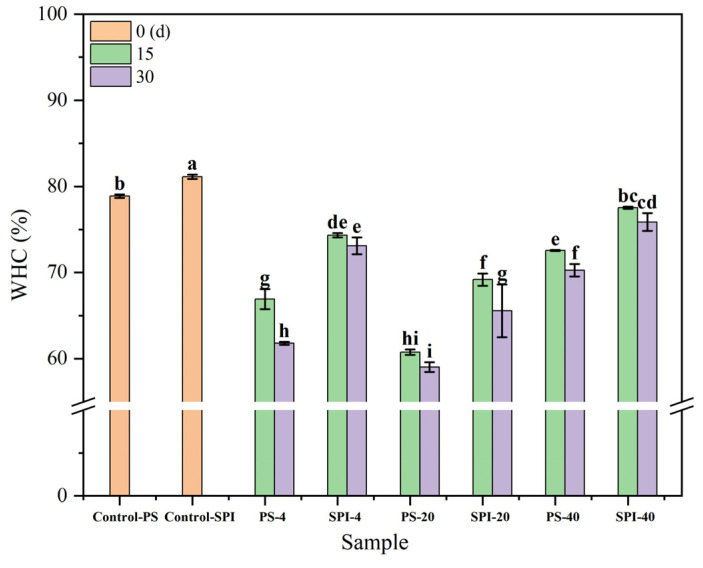
Effects of different cold storage times and temperatures on the WHC of surimi gel. There were significant differences between different letters in the figure (*p* < 0.05).

WHC is a crucial indicator for measuring the water binding capacity of surimi products. Figure 3 illustrates the trends in WHC changes of gels under different cold storage durations and temperatures. From 0 to 30 d, WHC storage in the control group was higher compared to the treated group. Both the control and treatment groups exhibited significant decreases in WHC over time (*p* < 0.05).

For the 4 °C storage, the WHC of the control group decreased significantly (*p* < 0.05) after 15 and 30 d of storage. In contrast, the WHC of the treatment group decreased gradually over the same period. Compared to the control group, the treatment group exhibited a slower rate of WHC decline during both 15 and 30 d of storage. This may be attributed to the addition of SPI, which enhanced the WHC of the composite gels by creating a denser gel structure with stronger water binding capabilities. These findings aligned with the research conducted by Wang et al. [28].

For the −20 °C storage, the WHC of the control group gels decreased significantly (*p* < 0.05) after 15 and 30 d of storage. For the treatment group, the WHC also showed a declining trend over the same periods of 15 and 30 d, but at a slower rate compared to the control group. Such improvement may be attributed to the addition of SPI, which improved the WHC of the composite gels. Besides, ice crystals with irregular shapes and sizes form under −20 °C may lead to protein denaturation [38], which significantly reduces the WHC of the gels (*p* < 0.05).

For the −40 °C storage, both the control group and the treatment group gels exhibited a declining trend in WHC after 15 and 30 d of storage. However, compared to the control group, the treatment group demonstrated a slower rate of WHC decline during both 15 and 30 d of cold storage at −40 °C, which may be due to the fact that ice crystals formed at −40 °C were smaller than those formed at −20 °C. Consequently, the rate of WHC decline was slower. Under the same conditions, the lower the freezing temperature and the shorter the storage duration, the slower the change in WHC was observed. The addition of SPI helped to form a denser gel network [39], which also protected proteins from hydrolysis [40]. Protein denaturation caused by frozen storage promoted the binding of myosin and actin, leading to myofibrillar protein contraction and subsequently reducing the WHC of the gels [41]. Such results aligned with the findings reported by Guo et al. [42]. The abovementioned results indicated that WHC stability was better at −40 °C compared to −20 °C, and SPI could enhance the WHC of surimi gels.

### 3.4. TPA

TPA is a commonly used analytical method for surimi products [43]. Table 1 summarizes the physical properties of gels under different cold storage durations and temperatures, including hardness, springiness, chewiness, and resilience. During the 0 to 30 d of cold storage, the treatment group exhibited an increasing trend in terms of hardness, springiness, and chewiness compared to the fresh samples of the control group. However, resilience exhibited a decreasing trend.

For the 4 °C storage, the hardness and chewiness of the control group and treatment group significantly increased (*p* < 0.05), with no significant change in springiness and a decline in resilience. Compared to the control group, the treatment group showed an increasing trend in hardness, springiness, and chewiness after 15 d of cold storage. By the 30 d of storage, hardness and chewiness continued to increase, while springiness remained almost unchanged. The resilience of the treatment group decreased slowly after 15 d of cold storage but increased slowly after 30 d of cold storage.

The increases in hardness and slower decreases in chewiness and resilience in the treatment group may be attributed to several factors. Firstly, protein oxidation and microbial influence could jointly affect the TPA properties [44]. Secondly, as storage duration increases, partial water loss contributes to the increase in gel hardness. The addition of SPI modifies the texture of the composite gels [19], potentially leading to the observed differences in physical properties between the control and treatment groups.

For the −20 °C storage, the hardness and chewiness of the control group and treatment group significantly increased (*p* < 0.05), while springiness and resilience decreased. Compared to the control group, the hardness of the treatment group increased significantly (*p* < 0.05) after 15 d of cold storage, but decreased slightly after 30 d. Elasticity, on the other hand, springiness remained relatively unchanged between 15 and 30 d of storage. Chewiness showed an increasing trend after 15 d of cold but then exhibited a declining trend by 30 d. In terms of resilience, the changes were similar between 15 and 30 d of cold storage.

These changes may be attributed to the formation of larger ice crystals at −20 °C, leading to significant water loss and protein denaturation [45]. Freezing and cold storage promoted the denaturation and aggregation of myosin, which affected the gel properties of frozen surimi [46]. The addition of SPI could enhance the WHC of the gel [42], thereby mitigating the formation of ice crystals and protein denaturation. This could explain the slower decreases in chewiness and resilience observed in the treatment group compared to the control group.

For the −40 °C storage, the control group and treatment group experienced a significant increase in hardness (*p* < 0.05) but a decrease in chewiness, springiness, and resilience (*p* < 0.05). Compared to the control group, the treatment group showed an increasing trend in hardness, springiness, and chewiness at both 15 and 30 d of cold storage. Specifically, the chewiness significantly increased at 15 d of cold storage, but the increase was more gradual at 30 d. Resilience remained unchanged at 15 d of storage but exhibited a slow increase trend after 30 d.

The addition of SPI under these storage conditions resulted in a higher textural stability of the composite gels, indicating that SPI altered the texture. This could be attributed to the smaller ice crystal size at −40 °C compared to −20 °C, and the prevention of SPI to protein hydrolysis [42], resulting in relatively less water loss. An et al. [46] found that the hardness of surimi products made from frozen fish mince increases during frozen storage due to protein damage and changes in myoglobin that favor crosslinking and gel formation, which was consistent with our findings.

Overall, under all three storage conditions, the treatment group exhibited less variation in gel texture and better stability compared to the control group. TPA stability was better at −40 °C compared to −20 °C, and the texture of SPI-added surimi gels was more stable. These observations aligned with the trends observed in ice crystal morphology, water distribution, and WHC.

### 3.5. Gel Strength

**Figure 4:**
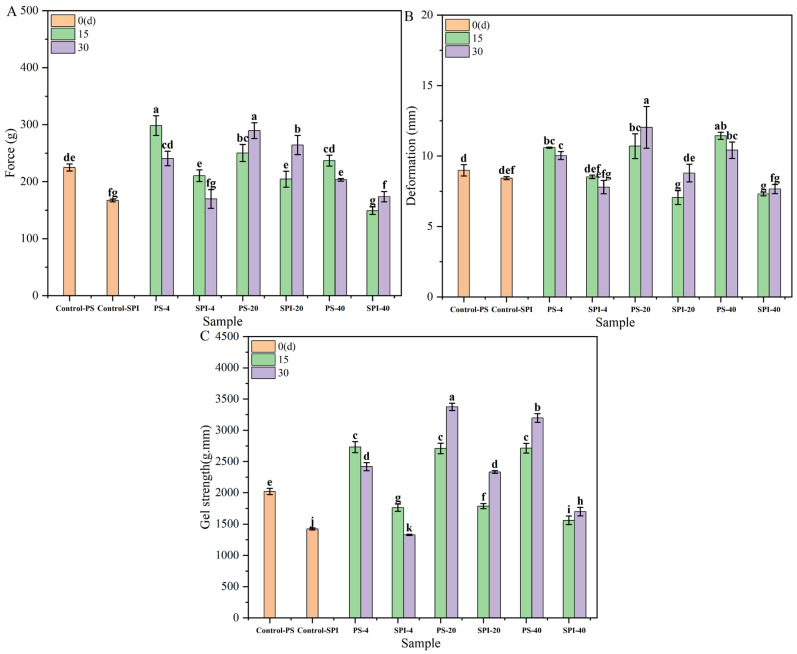
Gel strength values at different cold storage times and temperatures (*p* < 0.05). (**A**) force, (**B**) deformation, (**C**) gel strength. There are significant differences between different letters in the figure.

Figure 4 illustrates the changes in gel strength under different cold storage durations and temperatures on force, deformation and gel strength, respectively. Initially (0 d), the gel strength of the control group in fresh samples was 29.50% higher than that of the treatment group. Such a difference may be attributed to the reduction in gel strength caused by the addition of SPI [19].

For the 4 °C storage, the gel strength of the control group showed an upward trend after 15 and 30 d of storage. For the treatment group, the gel strength also increased after 15 d of cold storage but exhibited a slow downward trend after 30 d. Compared to the control group, the gel strength of the treatment group demonstrated a decreasing trend at both 15 and 30 d of cold storage. This may be attributed to the higher hardness of the treatment group, resulting in a smaller force value and shorter distance required for puncturing (Figure 4A,B). The changes in gel strength may be explained by several factors. Initially, during the early stages of cold storage, partial water was lost, leading to an increase in gel strength. However, as the storage duration increased, protein oxidation and microbial growth during cold storage may have contributed to a decrease in gel strength [44].

For the −20 °C storage, both the control group and the treatment group showed a statistically significant increase (*p* < 0.05) in gel strength. However, compared to the control group, the gel strength of the treatment group exhibited a downward trend after 15 and 30 d of cold storage. This difference may be attributed to several factors. Firstly, the formation of ice crystals during cold storage may lead to protein denaturation [47], which affects the gel structure and strength. Secondly, the significant loss of water content at these low temperatures may lead to a marked increase in gel strength (*p* < 0.05).

The addition of SPI appeared to slow down the rate of change in gel strength compared to the control group. This observation was consistent with the hypothesis that SPI may enhance the WHC of the gels, thus mitigating the effects of water loss and ice crystal formation. Furthermore, SPI had been shown to reduce the rate of ice crystal formation [23], which could explain the slower change in gel strength observed in the treatment group.

For the −40 °C storage, both the control group and the treatment group exhibited significant increases in gel strength (*p* < 0.05). Specifically, for both groups, the gel strength showed an upward trend after 15 and 30 d of cold storage. However, when compared to the control group, the gel strength of the treatment group demonstrated a decreasing trend at both 15 and 30 d of cold storage. The slower rate of change in gel strength observed in the treatment group may be attributed to the addition of SPI, which appeared to mitigate the effects of cold storage on the gel structure.

For the −40 °C storage, the formation of ice crystals was reduced, resulting in a slower rate of change in gel strength compared to −20 °C [23,45]. SPI addition further slowed down the rate of gel strength changes, indicating its protective effect on the gel structure.

Changes in myosin during frozen storage can inhibit the formation of cross-links and network structures during gelation, which may lead to a decrease in the mechanical properties of frozen surimi [48]. Across the three storage temperatures, the treatment group exhibited a smaller rate of change and more stable gel strength compared to the control group. Both the control and treatment groups showed better gel strength stability under −40 °C than at −20 °C. These observations aligned with the trends observed in ice crystal morphology, water distribution and WHC.

### 3.6. FTIR

FTIR spectroscopy is a commonly used method to determine protein conformation. Information regarding the secondary structure of proteins is derived from the amide I region of the spectrum (1700–1600 cm^−1^) [49]. Spectral bands corresponding to α-helix, random coil, β-sheet, and β-turn are typically located in the ranges of 1650–1663, 1638–1649, 1610–1637, and 1664–1690 cm^−1^, respectively. Table 2 presents the FTIR analysis of gels under different cold storage conditions.

Compared to the control group, the treatment group showed an increasing trend in α-helix, β-turn, and random coil content, while β-sheet content decreased in the fresh samples over the cold storage period of 0 to 30 d. This suggested that the addition of SPI altered the secondary structure of the gel.

For the 4 °C storage of 15 and 30 d, the α-helix, β-turn, and random coil content exhibited an increasing trend, while the β-sheet content showed a decreasing trend compared to the control group. In the treatment group after 15 d storage, the α-helix, β-turn, and random coil content decreased, while the β-sheet content increased. By the 30 d storage in the treatment group, the α-helix and β-turn content showed a slow increase, but the β-sheet and random coil content decreased. Compared to the control group, the treatment group showed an increasing trend in α-helix, β-sheet, and β-turn content, while random coil content decreased during both 15 and 30 d storage.

The results indicated that α-helix and random coil content decreased significantly, while β-sheet content increased significantly in the treatment group. This suggested that the gel structure in the treatment group was more stable, and the addition of SPI enhanced the secondary structure of the gel.

For the −20 °C storage, the α-helix, β-sheet, β-turn and random coil of the control group showed a slow downward trend after 15 d storage. After 30 d storage, α-helix, β-turn and random coil showed an increasing trend, and β-sheet showed a decreasing trend. The α-helix and the random coil of the treatment group showed a downward trend after 15 d storage, and the β-sheet and β-turn showed an increasing trend. After 30 d storage, α-helix showed an increasing trend, β-sheet, β-turn and random coil showed a decreasing trend. Compared with the control group, the α-helix and β-turn of the treatment group showed an increasing trend, while the β-sheet and random coil showed a decreasing trend at 15 and 30 d. The changes in α-helix, β-sheet, and β-turn in the treatment group were slower, and there was a significant decrease in the random coil. The gel structure of the treatment group was more stable, and the addition of SPI strengthened the secondary structure of the composite gel. The reason may be that the formation of ice crystals irreversibly denatures the protein, leading to changes in the secondary structure of the gel [50].

For the −40 °C storage, in the control group, α-helix, β-turn and random coil showed an increasing trend, and β-sheet showed a decreasing trend after 15 d storage. After 30 d storage, α-helix and β-sheet showed a downward trend, while β-turn and random coil showed an increasing trend. The α-helix and the random coil of the treatment group showed a downward trend after 15 d storage, and the β-sheet and β-turn showed an increasing trend. After 30 d storage, α-helix showed an increasing trend, β-sheet showed a decreasing trend, and β-turn and random coil showed no significant change. Compared with the control group, the α-helix and β-turn of the treatment group showed an increasing trend, and the β-sheet and random coil showed a decreasing trend after 15 d storage. After 30 d storage, α-helix, β-turn and random coil showed an increasing trend, and β-sheet showed a decreasing trend. During the 30 d storage, the α-helix in the treatment group increased, while β-sheet and β-turn showed slower changes, and the random coil remained basically unchanged, indicating a more stable gel structure. This could be due to the fact that cold storage at −40 °C produces fewer ice crystals and results in lower protein denaturation compared to −20 °C [2]. 

The decrease in α-helix structure was mainly due to structural changes in the myosin tail during frozen storage [51]. Gels with higher β-sheet content had better mechanical properties than those with higher α-helix content [28], which was consistent with the observed changes in gel strength. Short-term freezing had a smaller impact on quality attributes but could also lead to the unfolding of the myoglobin structure, resulting in a decrease in α-helix content and exposure of hydrophobic residues [46]. However, long-term freezing may lead to quality defects [52], including decreased protein solubility and WHC, which were the result of lipid oxidation and ice crystal formation [53]. The results suggested that the addition of SPI enhanced the structural stability of surimi gels.

### 3.7. Determination of Sulfhydryl Group Content

**Figure 5:**
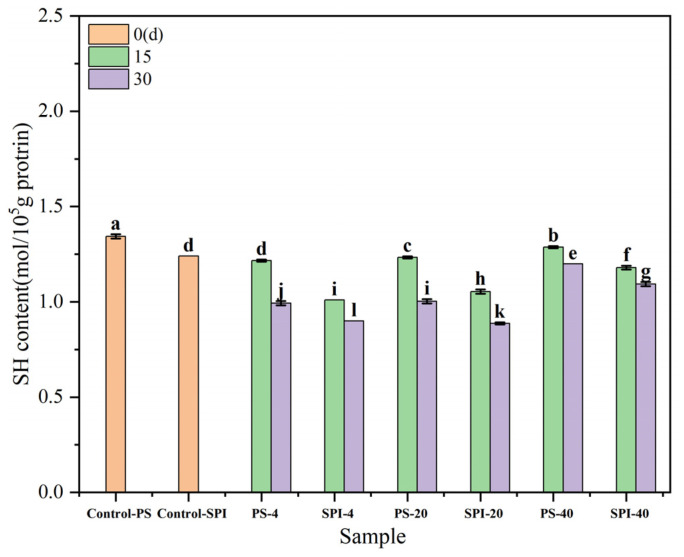
The effects of different cold storage times and temperatures on the sulfhydryl content of surimi gel. There were significant differences between different letters in the figure (*p* < 0.05).

The changes in total sulfhydryl content are related to the degree of protein oxidation. Figure 5 shows the trends in total sulfhydryl content in gels under different cold storage durations and temperatures. Compared to the control group, the total sulfhydryl content in the fresh samples of the treatment group exhibited a decreasing trend over the period of 0 to 30 d. This reduction could be attributed to the addition of SPI, which lowered the total sulfhydryl content.

For the 4 °C storage, the total sulfhydryl content in both the control and treatment groups showed a declining trend after 15 d of cold storage, with a significant decrease (*p* < 0.05) observed after 30 d storage. Compared to the control group, the total sulfhydryl content in the treatment group also exhibited a downward trend at both 15 and 30 d storage. This decrease was likely due to changes in total sulfhydryl groups resulting from the combined effects of protein oxidation and microbial activity [44]. These results were similar to the trends observed in the changes in protein secondary structure.

For the −20 °C storage, the total sulfhydryl content in both the control and treatment groups showed a declining trend after 15 d storage, with a significant decrease (*p* < 0.05) observed after 30 d storage. Compared to the control group, the total sulfhydryl content in the treatment group also exhibited a downward trend at both 15 and 30 d storage. The reason for these decreases could be attributed to the formation of numerous ice crystals and significant water loss during low-temperature storage, leading to irreversible denaturation of proteins [54]. This denaturation process can affect the sulfhydryl groups, resulting in a significant reduction in their total content (*p* < 0.05). The observed changes in sulfhydryl content were indicative of the oxidative status and stability of the protein during cold storage, which was crucial for maintaining the quality and functionality of surimi gels.

For the −40 °C storage, the total sulfhydryl content in both the control and treatment groups showed a declining trend after 15 and 30 d storage. However, when compared to the control group, the total sulfhydryl content in the treatment group exhibited a slower rate of decline at both 15 and 30 d storage. The reason for these relatively smaller changes in sulfhydryl content could be attributed to the fact that fewer ice crystals were formed at −40 °C compared to −20 °C, resulting in a slower rate of protein denaturation. This slower denaturation process had a less pronounced effect on the sulfhydryl groups, leading to smaller decreases in their total content.

Zhao et al. [55] found that SPI promoted the exposure of functional termini in surimi proteins, leading to an increase in the content of disulfide bonds. Hu et al. [52] found that the content of free sulfhydryl groups in SPI showed a downward trend as the freezing temperature decreased. This reduction in sulfhydryl content could be attributed to the recrystallization of ice or the redistribution of water during freezing, which caused the free sulfhydryl groups on the protein surface to lose their support and form disulfide bonds or other interactions. These findings were consistent with the results reported by An et al. [46], who observed a significant decrease in free sulfhydryl (-SH) content in fish myosin after 15 d of storage at −18 °C. 

### 3.8. Chemical Forces

**Figure 6:**
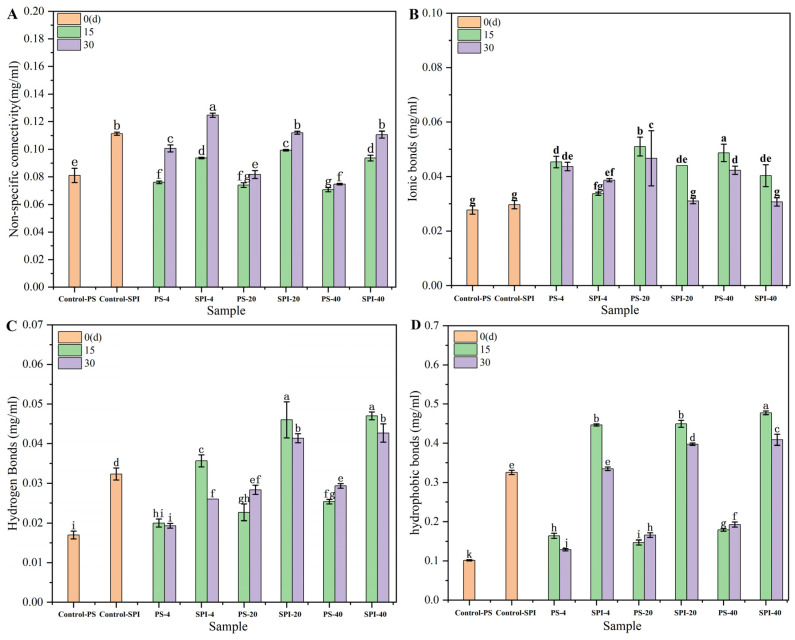
The effects of different cold storage times and cold storage temperatures on the chemical bonds of surimi gel. (**A**) nonspecific binding interactions, (**B**) ionic bonds, (**C**) hydrogen bonds, (**D**) hydrophobic interactions. There were significant differences between different letters in the figure (*p* < 0.05).

During the cold storage period, the chemical forces that governed the conformational stability of surimi gels exhibited specific trends as depicted in Figure 6. Ionic bonds and hydrogen bonds maintained protein-water interactions, while hydrophobic interactions and covalent bonds sustained protein-protein interactions [56].

Compared fresh samples to the control group, the treated gel samples exhibited an increasing trend in non-specific binding (Figure 6A) and ionic bonds (Figure 6B), while hydrogen bonds (Figure 6C) and hydrophobic interactions (Figure 6D) showed significant increases (*p* < 0.05).

For the 4 °C storage, the non-specific binding of the control group and treatment group showed a downward trend after 15 d of cold storage. The hydrogen bonds, ionic bonds and hydrophobic interactions showed an increasing trend. Nonspecific binding interactions, hydrogen bonds, ionic bonds and hydrophobic interactions showed an increasing trend after 30 d of cold storage in the control group. After 30 d of cold storage, non-specific binding, ionic bond and hydrophobic interaction of the treatment group showed an increasing trend, hydrogen bond showed a decreasing trend. Compared to the control group, the non-specific binding and hydrogen bonding of the treatment group showed an increasing trend at 15 and 30 d, the ionic bond increased slowly, and the hydrophobic interaction increased significantly. The relatively slower changes in chemical forces in the treatment group aligned with the trends observed in ice crystal morphology, water distribution, and WHC. The decrease in hydrogen bonds and increase in ionic bonds suggested the expansion and unfolding of α-helix structures [57], which could affect the conformational stability and functionality of the surimi gels. 

For the −20 °C storage, the control group exhibited specific trends in the non-specific binding interactions, hydrogen bonds, ionic bonds, and hydrophobic interactions over a 30 d storage. After 15 d storage, the non-specific binding showed a downward trend, and the hydrogen bond, ionic bond and hydrophobic interaction showed an increasing trend. After 30 d storage, the non-specific binding showed a slow increase, and the hydrogen bond, ionic bond and hydrophobic interaction increased significantly.

The non-specific binding of the treatment group showed a downward trend after 15 d storage, and the hydrogen bond, ionic bond and hydrophobic interaction showed an increasing trend. Non-specific binding, hydrogen bond, ionic bond and hydrophobic interaction showed an increasing trend after 30 d storage. Compared with the control group, the non-specific connection, hydrogen bond and hydrophobic bond of the treatment group showed an increasing trend, and the ionic bond showed a decreasing trend after 15 and 30 d storage.

These changes can be attributed to the formation of irregular ice crystals during cold storage, leading to irreversible protein denaturation [23]. This denaturation resulted in significant alterations in intermolecular forces (*p* < 0.05). The slower changes observed in the treatment group were likely due to the addition of SPI, which enhanced the WHC of the gels [42]. SPI may help stabilize the protein structure, mitigate the effects of cold storage on intermolecular interactions and thus preserve the conformational stability and functionality of the surimi gels. 

For the −40 °C storage, both the control group and the treatment group exhibited significant changes in intermolecular forces within surimi gels. In the control group, after 15 d storage, the non-specific binding exhibited a decreasing trend, while hydrogen bonding, ionic interactions, and hydrophobic interactions showed an increasing trend. This indicated that at lower temperatures, specific interactions between protein molecules are enhanced, particularly hydrogen bonding, ionic interactions, and hydrophobic interactions, which contribute to maintaining the stability and structural integrity of proteins. After 30 d storage, non-specific binding decreased slightly, but hydrogen bonding, ionic interactions, and hydrophobic interactions continued to increase, suggesting that these interactions continue to strengthen over extended periods of cold storage.

The treatment group exhibited similar trends but with slight moderation under the same cold storage conditions. After 15 d storage, the non-specific binding in the treatment group showed a decreasing trend, while hydrogen bonding, ionic interactions, and hydrophobic interactions increased. At 30 d storage, the non-specific binding remained almost unchanged, while hydrogen bonding, ionic interactions, and hydrophobic interactions continued to increase steadily.

Compared to the control group, the treatment group exhibited increased non-specific binding and more significant increases in hydrogen bonds, ionic bonds, and hydrophobic interactions at both 15 and 30 d. This suggested that certain components or additives in the treatment group may have enhanced these intermolecular interactions, thereby contributing to maintaining gel stability and quality during cold storage.

It is noteworthy that the changes in hydrophobic interactions, ionic bonds, and hydrogen bonds were relatively small during cold storage at −40 °C and −20 °C, further emphasizing the importance of these interactions in maintaining protein structure and function. Moreover, the significantly higher content of these interactions in the composite gels indicated a crucial role in gel formation and stability [58].

In conclusion, surimi gels underwent significant changes in intermolecular forces under storage with hydrophobic interactions, ionic bonds, and hydrogen bonds played a vital role in maintaining gel stability and quality. By comparing data from the control and treatment groups, it can be inferred that certain components or additives in the treatment group may help enhance these interactions, thus improving the cold storage stability of the gels.

### 3.9. Dynamic Rheological Properties

**Figure 7:**
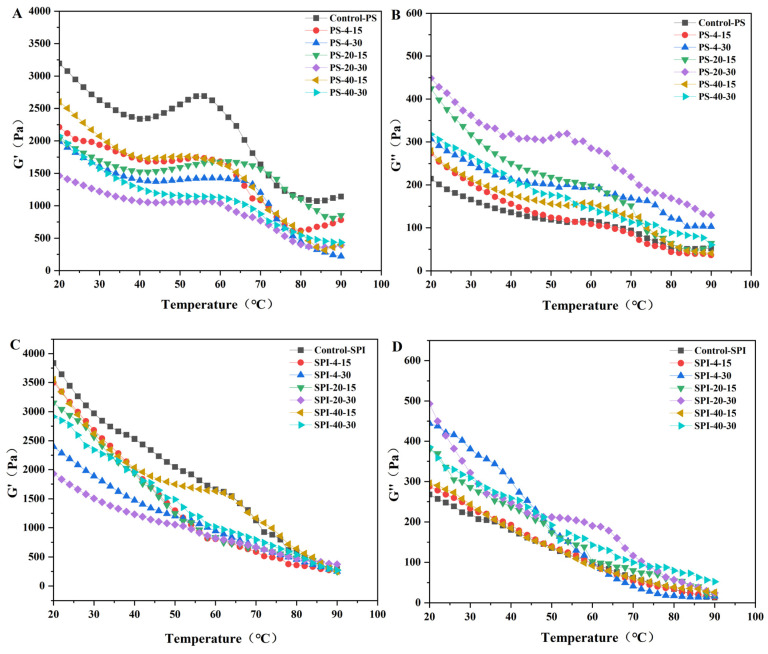
Effects of storage modulus (G′) and loss modulus (G″) of surimi gel during heating at different cold storage times and temperatures. PS indicates pure surimi group and SPI indicates SPI-surimi composite gel group. (**A**,**B**) show the storage G′ and G″ of surimi gel under different chilled storage conditions, respectively. (**C**,**D**) show the storage G′ and G″ of SPI-surimi composite gel under different chilled storage conditions, respectively.

Figure 7 illustrates the storage modulus (G′) and loss modulus (G″) of gels under different cold storage conditions. During thermal gelation, G′ and G″ characterize the formation of protein gel networks and are crucial indicators for assessing gel elasticity and viscosity [57]. G′ represents the energy storage capacity of the protein gel network, with higher values indicating better gel elasticity. G″ represents the energy lost as heat, reflecting the viscosity ratio and higher values suggest higher gel viscosity [54].

In all samples, the storage modulus (G′) was significantly larger than the loss modulus (G″), indicating their elastic and gel-like properties. Additionally, a higher G″ was associated with better WHC [59]. During cold storage, the G′ values of the fresh control samples (Figure 6A) initially decreased, reaching a low point at around 40 °C, then increased to a high point at approximately 55 °C, followed by a significant decrease, and a slight increase at around 80 °C. The G″ values (Figure 6B) showed a similar trend, with an initial decrease, a slight increase at around 60 °C, and then a decrease.

In the treated group, both G′ and G″ values decreased significantly. Compared to the control group, the G′ values of the treated group (Figure 6C) remained higher until 40 °C, indicating the formation of a denser and more uniform gel structure [6]. After 50 °C, the G″ values (Figure 6D) decreased. The dissociation of light chain myosin subunits around 50 °C led to an increase in viscosity, resulting in a decrease in G′. When the temperature exceeded 60 °C, heavy chain myosin and actin began to denature, forming a thermally irreversible gel network, leading to an increase in G′ [60].

The G′ values were highest and G″ values were lowest for both the control and treated groups in fresh samples. After storage for 15 d at −40 °C and 4 °C, the changes in G′ and G″ values were relatively small. Compared to the control group, the treated group showed minimal changes in G′ and G″ values after 30 d of cold storage at −40 °C. A possible reason for this may be due to the addition of SPI, which altered the quality of the composite gel [19]. The addition of SPI to the gel during cold storage resulted in minimal changes in G′ and G″ values. The trends observed in the changes of G′ and G″ values were consistent with the results of the WHC mentioned earlier.

The stability of the gel structure under different cold storage conditions suggested that SPI played a significant role in enhancing the gel’s resistance to structural degradation during cold storage. SPI may interact with the protein components of the gel, improving its stability and maintaining its viscoelastic properties over time. This interaction could also contribute to the better WHC observed in the treated group, as SPI may help retain water within the gel matrix.

Overall, the results indicated that SPI addition to the gel formulation may enhance its stability and maintain its desired functional properties under cold storage conditions. This was beneficial for the food industry, as it allowed for longer shelf life and better product quality for gel-based food products.

### 3.10. Color

Table 3 presents the differences in the whiteness of gels under various storage conditions. Compared to the fresh samples and the control group, the treatment group exhibited a downward trend in the L* value, while the a* and b* values showed an increasing trend, resulting in a decrease in whiteness. The addition of SPI reduced the whiteness of surimi gels [19].

For the 4 °C storage, the control group showed a consistent downward trend in L* value, a* value, b* value, and whiteness over 15 and 30 d of cold storage. As the storage time increased, the declining trends became more pronounced in the control group. In contrast, the treatment group exhibited a similar downward trend in L* value, b* value, and whiteness over 15 and 30 d of cold storage. However, for a* value, the treatment group showed an increasing trend, which became more significant with longer storage time. Compared to the control group, the treatment group exhibited a declining trend in L* value and whiteness, while showing an increasing trend in a* value and b* value after 15 and 30 d of cold storage. The slower change in whiteness observed in the treated group with SPI addition may be attributed to the consistent gel strength and texture mentioned earlier.

For the −20 °C storage, the L* value, a* value and whiteness of the control group showed a downward trend, and the b* value showed an upward trend. The L* value, a* value, b* value and whiteness of cold storage 30 d all showed a downward trend. The L* value, b* value and whiteness of the treated group showed a downward trend, and the a* value showed an upward trend. The L* value and whiteness of cold storage 30 d showed a downward trend, and the a* value and b* value showed an upward trend. The longer the storage time, the greater the change trend. Compared to the control group, the treatment group exhibited the following trends in L* value, whiteness, a* value, and b* value after 15 and 30 d of cold storage. The results were consistent with the study of Luo et al. [19]. The addition of SPI reduced the whiteness of the composite gel. This may be attributed to the formation of larger ice crystals during freezing at −20 °C, which disrupted the protein structure [61]. The significant decrease in whiteness observed was also consistent with the findings reported by Oh et al. [62].

For the −40 °C storage, the L* value, a* value and whiteness of the control group showed a downward trend, and the b* value showed an upward trend. The L* value, b* value and whiteness of the treated group showed a downward trend, and the a* value showed an upward trend. After 30 d of cold storage, L* value, a* value, b* value and whiteness all showed a downward trend. Compared with the control group, the L* value of the treatment group decreased significantly and the a* value increased significantly after 15 d of cold storage (*p* < 0.05). The b* value and whiteness showed a downward trend. The L* value, a and b* values of the treatment group were higher than those of the control group, but the whiteness was lower than that of the control group.

The addition of SPI reduced the whiteness of the composite gel, which may be due to the significant decrease in the whiteness of SPI gel after cold storage. This finding was consistent with previous research by Luo et al. [19]. Notably, under the same cold storage time, the decrease in gel whiteness was slower at lower temperatures, indicating that lower temperatures were more conducive to preserving the color of surimi gels.

### 3.11. SDS-PAGE

**Figure 8:**
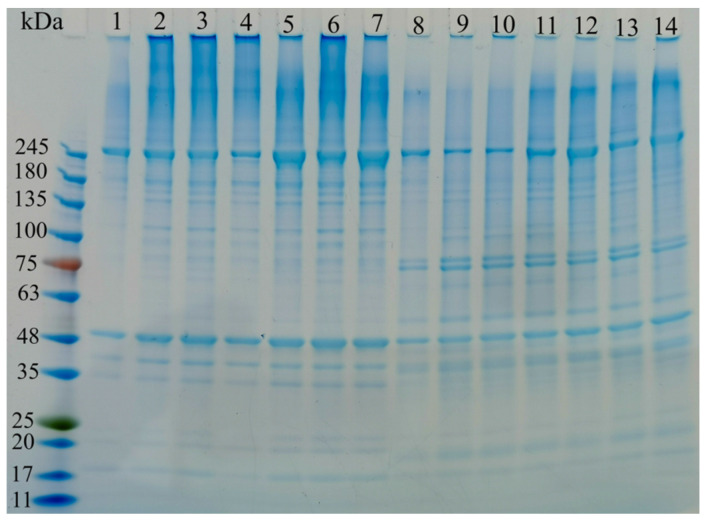
SDS-PAGE profiles of surimi gels at different cold storage times and temperatures. Number 1 represents Control-PS, number 2 represents PS-4-15, number 3 represents PS-4-30, number 4 represents PS-20-15, number 5 represents PS-20-30, number 6 represents PS-40-15, number 7 represents PS-40-30, number 8 represents Control-SPI, number 9 represents SPI-4-15, number 10 represents SPI-20-30, number 11 represents SPI-20-15, number 12 represents SPI-20-30, number 13 represents SPI-40-15, number 14 represents SPI-40-30.

Figure 8 presents the SDS-PAGE analysis of gel samples under different cold storage conditions. The changes in myofibrillar proteins within surimi gels after cold storage had a significant impact on product quality [63]. The SDS-PAGE analysis revealed that compared to fresh samples (lanes 1 and 8 in Figure 8), the MHC and actin bands in cold storage samples (lanes 2, 3, 4, 5, 6, and 7 in Figure 8, as well as lanes 9, 10, 11, 12, 13, and 14) exhibited minimal changes.

However, when comparing the control group (lanes 1, 2, 3, 4, 5, 6, and 7 in Figure 8) with the treated group (lanes 8, 9, 10, 11, 12, 13, and 14), the MHC and actin bands in the SPI-treated samples were significantly weakened. This suggested that the addition of SPI may lead to degradation or increased solubility of MHC and actin (within the 48–75 kDa range), resulting in reduced intensity of the protein bands. This finding aligns with previous research conducted by Zhang et al. [64].

As Figure 8 illustrates, the longer the cold storage time, the faster the protein changes occur. Conversely, lower cold storage temperatures resulted in slower protein changes, indicating that colder conditions were more conducive to preserving the gel’s integrity during storage. 

## 4. Conclusions

This study mainly investigated the effect of SPI on the gel quality of silver carp surimi under different storage conditions. Results found that SPI could effectively inhibit the growth of ice crystals in surimi gels, alter the water distribution, enhance the WHC, and strengthen the chemical interactions between surimi and SPI. The 10% SPI-added surimi demonstrated superior performance in terms of secondary structure, chemical interactions, and rheological properties compared to the control group during cold storage, with the best results observed at −40 °C. Additionally, SPI significantly increased the gel strength of surimi gels during cold storage, and the changes in gel strength were less pronounced in the treated group compared to the control. However, it is worth noting that the sulfhydryl groups and color of the treated group decreased more significantly during cold storage. Nevertheless, as the cold storage time increased, the inhibitory effect of the treated group was superior to that of the control. The shorter the cold storage time of the gel, the slower the quality decline. Under the same cold storage conditions, the SPI- surimi composite gel maintained better quality during cold storage. In summary, this study provides a theoretical basis for the application of SPI in the cold storage of surimi products.

## Figures and Tables

**Table 1:** Effects of TPA parameters of surimi gel at different cold storage times and temperatures.

Samples	Temperature (°C)	Time (d)	Hardness (g)	Springiness (mm)	Chewiness (g)	Resilience
PS	Control-PS	0	946.76 ± 20.69 ^i^	0.96 ± 0.01 ^bc^	816.10 ± 27.82 ^ef^	0.61 ± 0.03 ^a^
	4	15	989.41 ± 11.37 ^hi^	0.96 ± 0.01 ^bc^	823.24 ± 53.35 ^ef^	0.59 ± 0.02 ^ab^
		30	1116.86 ± 17.96 ^e^	0.96 ± 0.03 ^bc^	970.82 ±32.20 ^c^	0.58 ± 0.02 ^ab^
	−20	15	1055.55 ± 44.19 ^fg^	0.97 ± 0.01 ^ab^	875.28 ± 34.06 ^e^	0.57 ± 0.01 ^b^
		30	1396.06 ± 61.76 ^b^	0.94 ± 0.01 ^c^	1081.62 ± 34.30 ^b^	0.50 ± 0.01 ^c^
	−40	15	989.37 ± 10.62 ^hi^	0.94 ± 0.03 ^c^	776.42 ±29.95 ^g^	0.58 ± 0.01 ^ab^
		30	1024.93 ± 26.18 ^fgh^	0.94 ± 0.01 ^c^	808.45 ± 12.85 ^f^	0.50 ± 0.01 ^c^
SPI	Control-SPI	0	1007.12 ± 29.74 ^ghi^	0.99 ± 0.01 ^a^	865.21 ± 33.72 ^ef^	0.58 ± 0.01 ^ab^
	4	15	1162.57 ± 31.80 ^e^	0.97 ± 0.03 ^ab^	951.11 ± 40.98 ^cd^	0.58 ± 0.04 ^ab^
		30	1209.30 ± 51.82 ^d^	0.96 ± 0.03 ^bc^	1020.14 ± 57.86 ^bc^	0.59 ± 0.01 ^ab^
	−20	15	1382.42 ± 51.88 ^c^	0.97 ± 0.01 ^ab^	1046.10 ± 28.00 ^a^	0.57 ± 0.01 ^b^
		30	1462.71 ± 23.64 ^a^	0.94 ± 0.02 ^c^	1098.24 ± 27.17 ^a^	0.50 ± 0.01 ^c^
	−40	15	1030.62 ± 14.12 ^fgh^	0.97 ± 0.01 ^ab^	842.09 ± 10.81 ^ef^	0.58 ± 0.01 ^ab^
		30	1071.46 ± 12.25 ^ef^	0.96 ± 0.01 ^bc^	848.57 ± 6.90 ^ef^	0.51 ± 0.01 ^c^

Note: Different superscripts in the same column indicated significant differences (*p* < 0.05).

**Table 2:** Effects of different cold storage time and temperature on the secondary structure of surimi gel (%).

Samples	Temperature (°C)	Time (d)	α-Helix	β-Sheet	β-Turn	Random Coil
PS	Control-PS	0	18.23 ± 0.22 ^g^	45.49± 0.55 ^a^	9.89 ± 0.20 ^e^	26.38 ± 0.14 ^cd^
	4	15	18.74 ± 0.00 ^e^	43.81 ± 0.03 ^d^	10.39 ± 0.09 ^d^	27.05 ± 0.06 ^b^
		30	19.25 ± 0.02 ^c^	42.63 ± 0.01 ^e^	11.06 ± 0.03 ^abc^	27.04 ± 0.03 ^b^
	−20	15	18.20 ± 0.01 ^g^	45.27 ± 0.01 ^a^	10.07 ± 0.00 ^de^	26.44 ± 0.00 ^cd^
		30	18.77 ± 0.19 ^e^	43.58 ± 0.50 ^d^	10.25 ± 0.18 ^d^	27.38 ± 0.13 ^a^
	−40	15	18.50 ± 0.01 ^f^	44.61 ± 0.11 ^b^	10.26 ± 0.16 ^d^	26.62 ± 0.07 ^c^
		30	18.91 ± 0.08 ^de^	44.48 ± 0.42 ^bc^	10.09 ± 0.03 ^de^	26.50 ± 0.32 ^cd^
SPI	Control-SPI	0	19.49 ± 0.05 ^b^	42.90 ± 0.23 ^e^	10.95 ± 0.30 ^bc^	26.65 ± 0.13 ^c^
	4	15	18.81 ± 0.05 ^e^	44.10 ± 0.04 ^bcd^	10.74 ± 0.03 ^c^	26.34 ± 0.01 ^cd^
		30	19.51 ± 0.15 ^b^	42.81 ± 0.10 ^e^	11.29 ± 0.04 ^a^	26.37 ± 0.08 ^cd^
	−20	15	18.87 ± 0.13 ^e^	43.92 ± 0.03 ^cd^	10.98 ± 0.13 ^abc^	26.22 ± 0.06 ^d^
		30	19.76 ± 0.12 ^a^	42.79 ± 0.29 ^e^	10.85 ± 0.22 ^bc^	26.58 ± 0.06 ^c^
	−40	15	19.13 ± 0.04 ^cd^	44.44 ± 0.03 ^bc^	11.17 ± 0.07 ^ab^	25.24 ± 0.00 ^e^
		30	19.83 ± 0.15 ^a^	42.64 ± 0.13 ^e^	10.95 ± 0.12 ^bc^	26.65 ± 0.23 ^c^

Note: Different superscripts in the same column indicated significant differences (*p* < 0.05).

**Table 3:** Effects of different cold storage times and temperatures on the color of surimi gel.

Samples	Temperature (°C)	Time (d)	L*	a*	b*	Whiteness
PS	Control-PS	0	89.33 ± 0.88 ^a^	7.03 ± 0.06 ^d^	19.92 ± 0.18 ^e^	76.31 ± 0.06 ^a^
	4	15	87.84 ± 0.11 ^c^	6.61 ± 0.11 ^e^	19.73 ± 0.07 ^e^	75.89 ± 0.06 ^c^
		30	86.52 ± 0.13 ^d^	6.59 ± 0.02 ^e^	19.75 ± 0.07 ^e^	75.19 ± 0.08 ^d^
	−20	15	86.55 ± 0.19 ^d^	5.74 ± 0.16 ^g^	20.91 ± 0.35 ^d^	74.43 ± 0.04 ^e^
		30	84.28 ± 0.34 ^e^	5.71 ± 0.16 ^g^	19.79 ± 0.05 ^e^	74.11 ± 0.11 ^f^
	−40	15	88.89 ± 0.34 ^ab^	6.49 ± 0.21 ^e^	20.10 ± 0.13 ^e^	76.13 ± 0.03 ^ab^
		30	88.23 ± 0.39 ^bc^	6.07 ± 0.08 ^f^	20.06 ± 0.06 ^e^	75.95 ± 0.05 ^bc^
SPI	Control-SPI	0	83.56 ± 0.82 ^e^	7.93 ± 0.16 ^c^	23.85 ± 0.13 ^b^	69.92 ± 0.16 ^g^
	4	15	82.25 ± 0.24 ^f^	8.24 ± 0.09 ^ab^	22.92 ± 0.13 ^c^	69.86 ± 0.01 ^g^
		30	81.61 ± 0.18 ^f^	8.25 ± 0.01 ^ab^	22.88 ± 0.07 ^c^	69.51 ± 0.04 ^h^
	−20	15	78.92 ± 0.61 ^h^	8.07 ± 0.17 ^bc^	23.46 ± 0.92 ^b^	67.44 ± 0.15 ^k^
		30	77.72 ± 0.50 ^i^	8.38 ± 0.13 ^a^	23.94 ± 0.25 ^a^	66.23 ± 0.21 ^k^
	−40	15	80.56 ± 0.51 ^g^	8.07 ± 0.20 ^bc^	23.65 ± 0.37 ^b^	68.32 ± 0.17 ^i^
		30	79.83 ± 0.19 ^g^	7.86 ± 0.12 ^c^	23.58 ± 0.18 ^b^	68.01 ± 0.11 ^j^

Note: Different superscripts in the same column indicated significant differences (*p* < 0.05).

## Data Availability

The original contributions presented in the study are included in the article, further inquiries can be directed to the corresponding authors.

## References

[1] Park J.W. (2005). Surimi and Surimi Seafood.

[2] Jia R., Katano T., Yoshimoto Y., Gao Y., Watanabe Y., Nakazawa N., Osako K., Okazak E. (2018). Sweet potato starch with low pasting temperature to improve the gelling quality of surimi gels after freezing. Food Hydrocoll..

[3] Walayat N., Xiong H., Xiong Z., Moreno H.M., Nawaz A., Niaz N., Randhawa M.A. (2022). Role of Cryoprotectants in Surimi and Factors Affecting Surimi Gel Properties: A Review. Food Rev. Int..

[4] Sun X., Li Q., Ding N., Liang S., Mubango E., Zheng Y., Yu Q., Dai R., Tan Y., Luo Y. (2023). Cryoprotective effect of fistular onion stalk polysaccharide on frozen surimi derived from bighead carp: Physicochemical properties and gel quality during storage. Food Hydrocoll..

[5] Zhang Y., Zhang J., Chen Q., He N., Wang Q. (2022). High-Moisture Extrusion of Mixed Proteins from Soy and Surimi: Effect of Protein Gelling Properties on the Product Quality. Foods.

[6] Li X., Fan M., Huang Q., Zhao S., Xiong S., Yin T., Zhang B. (2022). Effect of micro- and nano-starch on the gel properties, microstructure and water mobility of myofibrillar protein from grass carp. Food Chem..

[7] Eddin A.S., Adegoke S., Issa A.T., Wilson C., Tahergorabi R. (2020). Physicochemical Changes of Surimi Gels with Addition of Different Particle Sizes of Citrus Peel Fiber. J. Aquat. Food Prod. Technol..

[8] Cao Y., Zhao L., Huang Q., Xiong S., Yin T., Liu Z. (2022). Water migration, ice crystal formation, and freeze-thaw stability of silver carp surimi as affected by inulin under different additive amounts and polymerization degrees. Food Hydrocoll..

[9] Hasanpour F., Hoseini E., Motalebi A.A., Darvish F. (2012). Effects of Soy protein concentrate and Xanthan gum on physical properties of Silver carp (*Hypophthalmichthys molitrix*) surimi. Iran. J. Fish. Sci..

[10] Zhang S., Ramaswamy H.S., Hu L., Ren J., Zhu S., Yu Y. (2023). Effect of pressure-shift freezing treatment on gelling and structural properties of grass carp surimi. Innov. Food Sci. Emerg. Technol..

[11] Yang J., Jiang Y., Li M., Deng S., Gao Y., Hu Y. (2024). Quality change in Bombay duck (*Harpadon nehereus*) surimi during frozen storage using different freezing methods. J. Texture Stud..

[12] Herrero A.M., Carmona P., Cofrades S., Jimenez-Colmenero F. (2008). Raman spectroscopic determination of structural changes in meat batters upon soy protein addition and heat treatment. Food Res. Int..

[13] Yue X., Li M., Liu Y., Zhang X., Zheng Y. (2021). Microbial diversity and function of soybean paste in East Asia: What we know and what we don’t?. Curr. Opin. Food Sci..

[14] Zheng L., Regenstein J.M., Zhou L.Y., Wang Z.J. (2022). Soy protein isolates: A review of their composition, aggregation, and gelation. Compr. Rev. Food Sci. Food Saf..

[15] Zhang T., Dou W., Zhang X., Zhao Y., Zhang Y., Jiang L., Sui X. (2021). The development history and recent updates on soy protein-based meat alternatives. Trends Food Sci. Technol..

[16] Liu H., Zhang H., Liu Q., Chen Q., Kong B. (2021). Filamentous myosin in low-ionic strength meat protein processing media: Assembly mechanism, impact on protein functionality, and inhibition strategies. Trends Food Sci. Technol..

[17] You S., Yang S., Li L., Zheng B., Zhang Y., Zeng H. (2022). Processing T echnology and Quality Change during Storage of Fish Sausages with T extured Soy Protein. Foods.

[18] Xu Y., Yu J., Xue Y., Xue C. (2023). Enhancing gel performance of surimi gels via emulsion co-stabilized with soy protein isolate and κ-carrageenan. Food Hydrocoll..

[19] Luo Y., Kuwahara R., Kaneniwa M., Murata Y., Yokoyama M. (2004). Effect of soy protein isolate on gel properties of Alaska pollock and common carp surimi at different setting conditions. J. Sci. Food Agric..

[20] Zhang H., Xiong Y., Bakry A.M., Xiong S., Yin T., Zhang B., Huang J., Liu Z., Huang Q. (2019). Effect of yeast β-glucan on gel properties, spatial structure and sensory characteristics of silver carp surimi. Food Hydrocoll..

[21] Wei Y., Zhang T., Yu F., Xue Y., Li Z., Wang Y., Xue C. (2018). Effects of curdlan on the texture and structure of Alaska pollock surimi gels treated at 120 °C. Int. J. Food Prop..

[22] Yang Y., Meng L., Wang Y., Yan B. (2022). Effects of exogenous lipids on gelling properties of silver carp surimi gel subjected to microwave heating. Food Sci. Nutr..

[23] Luo X., Huang K., Niu Y., Zhang X., An Y., Liu R., Xiong S., Hu Y. (2023). Effects of freezing methods on physicochemical properties, protein/fat oxidation and odor characteristics of surimi gels with different cross-linking degrees. Food Chem..

[24] Zhang C., Chen L., Lu M., Ai C., Cao H., Xiao J., Zhong S., Teng H. (2023). Effect of cellulose on gel properties of heat-induced low-salt surimi gels: Physicochemical characteristics, water distribution and microstructure. Food Chem. X.

[25] Zhao Y., Wei G., Li J., Tian F., Zheng B., Gao P., Zhou R. (2023). Comparative study on the effect of different salts on surimi gelation and gel properties. Food Hydrocoll..

[26] Jiang X., Chen Q., Xiao N., Du Y., Feng Q., Shi W. (2022). Changes in Gel Structure and Chemical Interactions of Hypophthalmichthys molitrix Surimi Gels: Effect of Setting Process and Different Starch Addition. Foods.

[27] Zhao Y., Li J., Wei G., Ying X., Zheng B., Gao P., Zhou R. (2023). Fortification of surimi gels by tuning the synergetic effect of multiple enzyme-related factors. Food Hydrocoll..

[28] Wang Y., Tu X., Shi L., Yang H. (2022). Quality characteristics of silver carp surimi gels as affected by okara. Int. J. Food Prop..

[29] Yang Y., Liu X., Xue Y., Xue C., Zhao Y. (2019). The process of heat-induced gelation in Litopenaeus vannamei. Food Hydrocoll..

[30] Bao Y., Wang K., Yang H., Regenstein J.M., Ertbjerg P., Zhou P. (2020). Protein degradation of black carp (*Mylopharyngodon piceus*) muscle during cold storage. Food Chem..

[31] Leygonie C., Britz T.J., Hoffman L.C. (2012). Impact of freezing and thawing on the quality of meat: Review. Meat Sci..

[32] Li N., Shen Y., Liu W., Mei J., Xie J. (2018). Low-Field NMR and MRI to Analyze the Effect of Edible Coating Incorporated with MAP on Qualities of Half-Smooth Tongue Sole (*Cynoglossus Semilaevis Günther*) Fillets during Refrigerated Storage. Appl. Sci..

[33] Li Q., Yi S., Wang W., Xu Y., Mi H., Li X., Li J. (2022). Different Thermal T reatment Methods and TGase Addition Affect Gel Quality and Flavour Characteristics of Decapterus maruadsi Surimi Products. Foods.

[34] Ding J., Zhao X., Li X., Huang Q. (2022). Effects of different recovered sarcoplasmic proteins on the gel performance, water distribution and network structure of silver carp surimi. Food Hydrocoll..

[35] Zhang Z., Regenstein J.M., Zhou P., Yang Y. (2017). Effects of high intensity ultrasound modification on physicochemical property and water in myofibrillar protein gel. Ultrason. Sonochem..

[36] Shen Z., Tian M., Wang F., Liu Y., Wu J., Li X. (2024). Enhancing freeze-thaw stability and flavor in surimi products: Impact of virgin coconut oil and fish oil incorporation. Food Biosci..

[37] Sun Q., Kong B., Liu S., Zheng O., Zhang C. (2021). Ultrasonic Freezing Reduces Protein Oxidation and Myofibrillar Gel Quality Loss of Common Carp (*Cyprinus carpio*) during Long-Time Frozen Storage. Foods.

[38] Walayat N., Tang W., Nawaz A., Ding Y., Liu J., Lorenzo J.M. (2022). Influence of Konjac oligo-glucomannan as cryoprotectant on physicochemical and structural properties of silver carp surimi during fluctuated frozen storage. LWT—Food Sci. Technol..

[39] Lin D., Zhang L., Li R., Zheng B., Rea M.C., Miao S. (2019). Effect of plant protein mixtures on the microstructure and rheological properties of myofibrillar protein gel derived from red sea bream (*Pagrosomus major*). Food Hydrocoll..

[40] Rawdkuen S., Benjakul S., Visessanguan W., Lanier T.C. (2007). Effect of chicken plasma protein and some protein additives on proteolysis and gel forming ability of sardine (*sardinella gibbosa*) surimi. J. Food Process. Preserv..

[41] Farouk M.M., Wieliczko K.J., Merts I. (2004). Ultra-fast freezing and low storage temperatures are not necessary to maintain the functional properties of manufacturing beef. Meat Sci..

[42] Guo J., Hu L., Yang X., Yu S., Liu Y., Jin Y. (2015). Influence of soy protein isolate prepared by phosphate-assisted hydrothermalcooking on the gelation of myofibrillar protein. J. Am. Oil Chem. Soc..

[43] Liu Y., Sun Q., Pan Y., Wei S., Xia Q., Liu S., Ji H., Deng C., Hao J. (2021). Investigation on the correlation between changes in water and texture properties during the processing of surimi from golden pompano (*Trachinotus ovatus*). J. Food Sci..

[44] Huang Q., Jiao X., Yan B., Zhang N., Huang J., Zhao J., Zhang H., Chen W., Fan D. (2022). Changes in physicochemical properties of silver carp (*Hypophthalmichthys molitrix*) surimi during chilled storage: The roles of spoilage bacteria. Food Chem..

[45] Liu S., Zeng X., Zhang Z., Long G., Lyu F., Cai Y., Liu J., Ding Y. (2020). Effects of Immersion Freezing on Ice Crystal Formation and the Protein Properties of Snakehead (*Channa argus*). Foods.

[46] An Y., Youa J., Xionga S., Yin T. (2018). Short-term frozen storage enhances cross-linking that was induced by transglutaminase in surimi gels from silver carp (*Hypophthalmichthys molitrix*). Food Chem..

[47] Banerjee R., Maheswarappa N.B. (2017). Superchilling of muscle foods: Potential alternative for chilling and freezing. Critical Reviews. Food Sci. Nutr..

[48] Benjakul S., Visessanguan W., Thongkaew C., Tanaka M. (2003). Comparative study on physicochemical changes of muscle proteins from some tropical fish during frozen storage. Food Res. Int..

[49] Wang K., Luo S., Cai J., Sun Q., Zhao Y., Zhong X., Jiang S., Zheng Z. (2015). Effects of partial hydrolysis and subsequent cross-linking on wheat gluten physicochemical properties and structure. Food Chem..

[50] Wu Y., Xu X., Sheng L. (2023). Effects of different plant proteins on the quality and characteristics of heat induced egg white protein gel under freezing conditions. Food Hydrocoll..

[51] Leelapongwattana K., Benjakul S., Visessanguan W., Howell N.K. (2005). Physicochemical and biochemical changes during frozen storage of minced flesh of lizardfish (*Saurida micropectoralis*). Food Chem..

[52] Hu H., Feng Y., Zheng K., Shi K., Yang Y., Yang C., Wang J. (2024). The effect of subzero temperatures on the properties and structure of soy protein isolate emulsions. Food Chem..

[53] Jenkelunas P.J., Li-Chan E.C. (2018). Production and assessment of Pacific hake (*Merluccius productus*) hydrolysates as cryoprotectants for frozen fish mince. Food Chem..

[54] Chen X., Li X., Yang F., Wu J., Huang D., Huang J., Wang S. (2022). Effects and mechanism of antifreeze peptides from silver carp scales on the freeze-thaw stability of frozen surimi. Food Chem..

[55] Zhao Y., Wei K., Chen J., Wei G., Li J., Zheng B., Song Y., Gao P., Zhou R. (2024). Enhancement of myofibrillar protein gelation by plant proteins for improved surimi gel characteristics: Mechanisms and performance. LWT—Food Sci. Technol..

[56] Zhang Z., Xiong Z., Lu S., Walayat N., Hu C., Xiong H. (2020). Effects of oxidative modification on the functional, conformational and gelling properties of myofibrillar proteins from Culter alburnus. Int. J. Biol. Macromol..

[57] Tian H., Chen X., Chen C., Wu J., Huang J., Zhao L., Wang S. (2022). Analysis of the shape retention ability of antifreeze peptide-based surimi 3D structures: Potential in freezing and thawing cycles. Food Chem..

[58] Lin J., Hong H., Zhang L., Zhang C., Luo Y. (2019). Antioxidant and cryoprotective effects of hydrolysate from gill protein of bighead carp (*Hypophthalmichthys nobilis*) in preventing denaturation of frozen surimi. Food Chem..

[59] Zhao Y., Zhou G., Zhang W. (2019). Effects of regenerated cellulose fiber on the characteristics of myofibrillar protein gels. Carbohydr. Polym..

[60] Yu N., Xu Y., Jiang Q., Xia W. (2016). Textural and physicochemical properties of surimi gels prepared with potassium and calcium chloride as substitutes for sodium chloride. Int. J. Food Prop..

[61] Nakazawa N., Okazaki E. (2020). Recent research on factors influencing the quality of frozen seafood. Fish. Sci..

[62] Oh J., Karadeniz F., Gao Y., Kim H., Kim S., Jung J., Cheon J., Kong C. (2018). Gel-Forming Ability of Surimi from Aquacultured Pagrus major as Affected by Freeze-Thaw Cycle. Turk. J. Fish. Aquat. Sci..

[63] Gao S., Zhuang S., Zhang L., Lametsch R., Tan Y., Li B., Hong H., Luo Y. (2023). Proteomic evidence of protein degradation and oxidation in brined bighead carp fillets during long-term frozen storage. Food Chem..

[64] Zhang Y., Puolanne E., Ertbjerg P. (2021). Mimicking myofibrillar protein denaturation in frozen-thawed meat: Effect of pH at high ionic strength. Food Chem..

